# The Gastric Microbiome Communities and Endoscopic Mucosal Morphologies Associated with Premalignant Conditions

**DOI:** 10.3390/microorganisms13112499

**Published:** 2025-10-30

**Authors:** Takuya Shijimaya, Tomomitsu Tahara, Tsubasa Shimogama, Jumpei Yamazaki, Sanshiro Kobayashi, Naohiro Nakamura, Yu Takahashi, Yusuke Honzawa, Takashi Tomiyama, Makoto Naganuma

**Affiliations:** 1 Third Department of Internal Medicine, Kansai Medical University, Hirakata 573-1191, Japan; shijimat@hirakata.kmu.ac.jp (T.S.); tsushimogama@yahoo.co.jp (T.S.); kobayass@hirakata.kmu.ac.jp (S.K.); nakamnao@hirakata.kmu.ac.jp (N.N.); takahyu@hirakata.kmu.ac.jp (Y.T.); honzaway@hirakata.kmu.ac.jp (Y.H.); tomiyata@hirakata.kmu.ac.jp (T.T.); naganuma@hirakata.kmu.ac.jp (M.N.); 2 Translational Research Unit, Veterinary Teaching Hospital, Faculty of Veterinary Medicine, Hokkaido University, Sapporo 060-0818, Japan; j.yamazaki@vetmed.hokudai.ac.jp; 3 One Health Research Center, Hokkaido University, Sapporo 060-0818, Japan

**Keywords:** gastric microbiome, gastric mucosa, narrow-band imaging, magnifying endoscopy, *Helicobacter pylori*, gastric cancer, 16S rRNA sequencing, DNA methylation

## Abstract

The risk of *Helicobacter pylori* (*H. pylori*)-related gastric tumorigenesis is closely associated with the degree of chronic gastritis, although other gastric mucosa microbes may be relevant in this process. The morphological identification of the gastric mucosa associated with the cancer-promoting microbiome may have important implications for gastric cancer prevention. This study characterized gastric mucosa microbiome communities in relation to their mucosal morphologies. A total of 94 biopsies from non-neoplastic gastric bodies underwent 16S rRNA sequencing. Microbiome structures were characterized in relation to their mucosal morphologies, which were obtained using narrow-band imaging with magnifying endoscopy. *H. pylori* infection- and inflammatory mucosa-associated gastric mucosal morphologies exhibited decreased bacterial alpha diversity measures and an increase in the abundance of the *Helicobacter* genus, while the mucosal morphology associated with severely atrophic mucosa exhibited increased bacterial alpha diversity measures and a decrease in the abundance of the *Helicobacter* genus. This type of mucosal morphology was also associated with increased levels of well-known gastric cancer-related bacteria, e.g., *Streptococcus*. The microbial dysbiosis associated with gastric mucosa morphology also correlated well with the occurrence of gastric cancer and the DNA methylation status. Our results suggest that gastric microbiome communities correlate well with their premalignant condition-associated mucosal morphologies.

## 1. Introduction

Infection with *Helicobacter pylori* (*H. pylori*) is an established risk for the development of gastric cancer and its premalignant conditions [1,2,3,4]. *H. pylori* contributes to gastric carcinogenesis by producing persistent acute-on-chronic inflammation in the gastric epithelium, leading to host somatic changes, such as epigenetic anomalies [5]. Importantly, gastric cancer risk varies considerably with the degree of *H. pylori*-related gastritis. Gastritis with severe neutrophil infiltration and damage to the surface epithelium may be associated with multifocal atrophic gastritis and is often observed in patients with gastric cancer [1]. However, completion of severe atrophic gastritis is associated with a decrease or disappearance of *H. pylori* [6], despite its higher risk of gastric cancer and host somatic changes like DNA methylation [7]. Recent metagenomic approaches have suggested a link between non-*H. pylori* microbiota and gastric tumorigenesis [8,9]. A case–controlled study that compared patients with gastric cancer with patients without cancer found that the gastric microbiome has potential cancer-promoting activities [8,9]. Indeed, it is proposed that changes occurring in the stomach because of decreased acid secretion as a result of chronic *H. pylori* infection allow the establishment of a new microbiota that contributes to malignant transformation through the maintenance of inflammation and nitrate conversion into N-nitrosamines [10,11]. It is possible that non-*H. pylori* bacteria may have a role in maintaining host genotoxic changes and promoting gastric tumorigenesis in the long-term outcome of *H. pylori* infection. It is reported that the degree of histological gastritis correlates with the gastric mucosal morphologies observed using magnifying endoscopy with narrow-band imaging (NBI) [12]. The NBI system uses narrow bands of red, blue, and green filters to visualize mucosal and capillary patterns clearly [13]. Combining magnifying endoscopy and the NBI system can more clearly visualize microscopic mucosal structures and their capillary patterns, which can better predict the histopathological state of endoscopic lesions throughout the gastrointestinal tract [14,15,16]. We have previously demonstrated that gastric mucosal morphologies visualized using magnifying NBI correlate well with the degree of *H. pylori*-related gastritis [12], gastric cancer occurrence [17], and DNA methylation anomalies [7], suggesting that gastric mucosal morphologies represent *H. pylori* infection-related gastric premalignant conditions. Morphological identification of the gastric mucosa associated with the cancer-promoting microbiome may also have important clinical implications for gastric cancer prevention. Therefore, we investigated the microbiome communities associated with the gastric mucosal morphologies observed via magnifying NBI endoscopy and assessed their correlation with the DNA methylation anomalies associated with gastric mucosal morphologies.

## 2. Materials and Methods

### 2.1. The Study Cohort

This study involved 94 participants (23 with gastric cancer and 71 without evidence of cancer) from a previous study of the relationship between gastric mucosal morphology and DNA methylation anomalies [7]. The patients with gastric cancer visited Fujita Health University Hospital for laparoscopic surgery or endoscopic mucosal dissection, while the other 71 participants underwent upper gastroscopy for various reasons, e.g., annual malignancy screening, secondary check-up after barium X-ray examination for suspected gastric diseases, and abdominal symptoms. Those with severe systemic disease or advanced chronic liver disease, those on non-steroidal anti-inflammatory drugs, proton-pump inhibitors, H2 receptor antagonists, or antibiotics, those with a history of *H. pylori* eradication therapy, and those with a history of gastrectomy were not included in the study cohort. The study’s protocol was approved by Fujita Health University School of Medicine and Kansai Medical University. All participants gave written informed consent for study enrolment and publication of identifying information/images. The study was conducted in accordance with the relevant guidelines and regulations.

### 2.2. Endoscopy, Classification of Gastric Mucosa Morphology, and Sample Collection for Histological and Molecular Analyses

**Figure 1:**
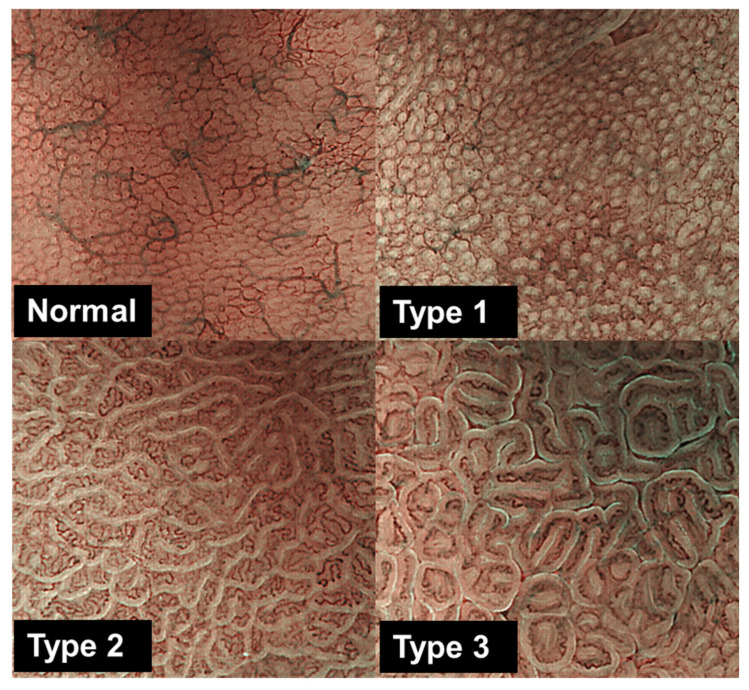
Characterization of gastric mucosa morphologies in the corpus seen with magnifying NBI. Endoscopy [12]. Normal: small, round pits surrounded by subepithelial capillary networks. Type 1: slightly enlarged, round pit with unclear or irregular subepithelial capillary networks. Type 2: obviously enlarged, oval or prolonged pit with increased density of irregular vessels. Type 3: well-demarcated, oval or tubulovillous pit with clearly visible coiled or wavy vessels.

For all participants, non-neoplastic mucosae of the gastric corpus, where gastric samples were obtained, were morphologically characterized during examination using magnifying NBI endoscopy [7]. All participants underwent esophagogastroduodenoscopy using a magnifying video endoscope (Olympus GIF-H260Z and a CV260SL/CV290SL endoscopic system, Olympus Medical Systems, Tokyo, Japan), and the non-neoplastic mucosae of the greater curvature in the gastric corpus were carefully observed using complete magnification coupled with an NBI light source. Based on our previous studies, gastric mucosa was morphologically categorized as normal (small and round pits with uniform subepithelial capillary networks), type 1 (slightly enlarged round pits with indistinct subepithelial capillary networks), type 2 (remarkably enlarged pits with irregular vessels), or type 3 (clearly demarcated oval or tubulovillous pits with bulky coiled or wavy vessels) (Figure 1) [12]. This classification system is reported to distinguish chronic gastritis histological degree clearly, and to correlate with gastric cancer occurrence and DNA methylation status [7,12,17]. The gastric mucosa morphological classification of each case was based on the most predominant magnifying NBI patterns, which were taken as endoscopic pictures. Biopsies were taken from the targeted sites. Based on the NBI patterns, of the 94 participants, 23 (24.5%), 15 (15.9%), 34 (36.2%), and 22 (23.4%) had normal, type 1, type 2, and type 3 gastric morphologies, respectively. At least two biopsies were obtained from the sites at which gastric mucosa morphologies were evaluated using endoscopy. Although the two biopsies were not from the same point, they were taken as close as possible within the targeted site. In patients with gastric cancer, the biopsies were obtained from sites that were sufficiently distant from cancerous lesions (<2 cm). One biopsy was fixed in 10% formalin and embedded in paraffin for histological examination. Hematoxylin and eosin staining was used to assess the degrees of neutrophil and mononuclear cell infiltration, atrophy, and metaplasia based on the updated Sydney system [18] and scored from 0 (normal) to 3 (marked). Neutrophil or mononuclear cell infiltration scores of ≥2 indicated inflammatory mucosa, while atrophy and metaplasia scores of ≥2 indicated atrophic mucosa. Genomic DNA was extracted from the other biopsy using the DNA precipitation method. Briefly, the specimen was incubated overnight at 55 °C in lysis buffer containing 25 mM EDTA (pH 8.0), 2% SDS, and proteinase K. The reaction solution was precipitated and then washed with isopropyl alcohol and 80% ethanol. Genomic DNA was suspended in TE buffer (pH 8.0), followed by quality evaluation through electrophoresis on a 1% agarose gel stained with ethidium bromide, which confirmed that the DNA samples were not degraded.

### 2.3. Detection of H. pylori Infection

*H. pylori* infection was detected through immunohistochemical analysis of the biopsies using an anti-*H. pylori* polyclonal antibody (Dako, Glostrup, Denmark), the ^13^C-urea breath test with UBIT tablets (Otsuka Pharmaceutical, Tokyo, Japan), and the serum anti-*H. pylori* IgG titer. A positive result from any of these tests indicated *H. pylori* infection, and a negative result from any of these tests was diagnosed as *H. pylori* negative.

### 2.4. 16S rRNA Gene Amplicon Sequencing

The biopsy DNA samples (10 ng per sample) underwent 16S rRNA gene amplification using the primers, 341F (5′TCGTCGGCAGCGTCAGATGTGTATAAGAGACAGCCTACGGGNGGCWGCAG) and 805R (GTCTCGTGGGCTCGGAGATGTGTATAAGAGACAGGACTACHVGGGTATCTAATCC), targeting the V3–V4 hypervariable region. The 5’ ends of the primers included Illumina’s universal adapter sequences. The following PCR program was used: denaturation at 95 °C for three minutes, 35 cycles at 95 °C for 30 s, 55 °C for 40 s, and 72 °C for 40 s, and a final elongation at 72 °C for 5 min.

The PCR products were then purified, and the Nextera XT Index kit (Illumina Inc., San Diego, CA, USA) was added, followed by an additional twelve cycles of PCR. Next, the DNA library concentrations were normalized, and the libraries were pooled for next-generation sequencing on a MiSeq platform (Illumina Inc.) using a MiSeq Reagent Kit version 3 (2 × 300 bp Paired-End Reads, Illumina Inc.). The sequencing results were processed using the Quantitative Insights into Microbial Ecology (QIIME) pipeline (version 2023.2). Primer-trimmed sequences were clustered to amplicon sequence variants using the q2-dada2 plugin, and sequences with anonymous bases and chimeras were filtered by cutting 30 bp upstream of the forward and reverse primer sequences with truncation lengths of 280 bp and 240 bp for the forward and reverse sequences, respectively. The amplicon sequence variant table in QIIME2 was used to calculate bacterial alpha diversity measures. The mean relative abundance (percentage among all reads) in each group was compared at the phylum, family, and genus levels.

### 2.5. DNA Methylation Analysis

Information about the DNA methylation status of the RORA, GDNF, PRDM5, MLF1, MYOD1, SLC16A12, IGF2, MIR124A1, and CDH1 genes was available for this cohort [7]. These genes were selected based on methylation frequency in gastric cancer (RORA, GDNF, PRDM5, and MLF1 [19]) or *H. pylori*-infected gastric mucosa (MYOD1, SLC16A12, IGF2, MIR124A1, and CDH1 [20]). Bisulfite pyrosequencing was used to evaluate the methylation status of bisulfite-treated genomic DNA, which was achieved using an EZ DNA Methylation Kit (Zymo Research, Irvine, CA, USA) as per the manufacturer’s protocol, with 500 ng of DNA being used per reaction. Pyrosequencing was performed using a PSQ24 system with a Pyro-Gold reagent Kit (QIAGEN, Tokyo, Japan), and the results were analyzed using the PyroMark Q24 software (QIAGEN). Pyrosequencing was done using our previously reported primers [7,20].

### 2.6. Statistical Analyses

Continuous variables were compared between two groups using the Mann–Whitney U test. Correlation between continuous variables in two groups was assessed using Spearman correlation analysis. *p* < 0.05 was considered statistically significant.

## 3. Results

### 3.1. The Clinicopathological Characteristics of the Study Cohort

The clinicopathological characteristics in relation to their gastric mucosal morphologies are shown in Supplementary Table S1. As we reported previously [7,12], the samples with normal patterns did not have *H. pylori* infection, gastric cancer, inflammatory mucosa, or atrophic mucosa. *H. pylori* infection was frequent in gastric mucosa morphology type 1 and type 2, while the prevalence of inflammatory mucosa was more frequent in type 2 than in type 1. The type 3 pattern was characterized as atrophic mucosa and was frequently observed in patients with gastric cancer. Typical gastric mucosa histological findings based on hematoxylin and eosin staining in relation to their NBI gastric mucosa patterns are shown in Figure S1. Additionally, a higher mean methylation Z score was observed in relation to mucosal patterns from normal to type 1, 2, and 3.

### 3.2. Bacterial Compositions Associated with Gastric Mucosal Morphology

All 94 gastric mucosa samples were available for 16S rRNA gene sequencing. A total of 1,179,841 sequences (mean: 12,551) were obtained after quality filtering. Operational taxonomic units were clustered based on 99% sequence similarity with at least 10 identical sequences, and assigned against the curated Greengenes v.13.8 reference database on the QIIME website (http://qiime.org/home_static/dataFiles.html: 27 October 2023).

**Figure 2:**
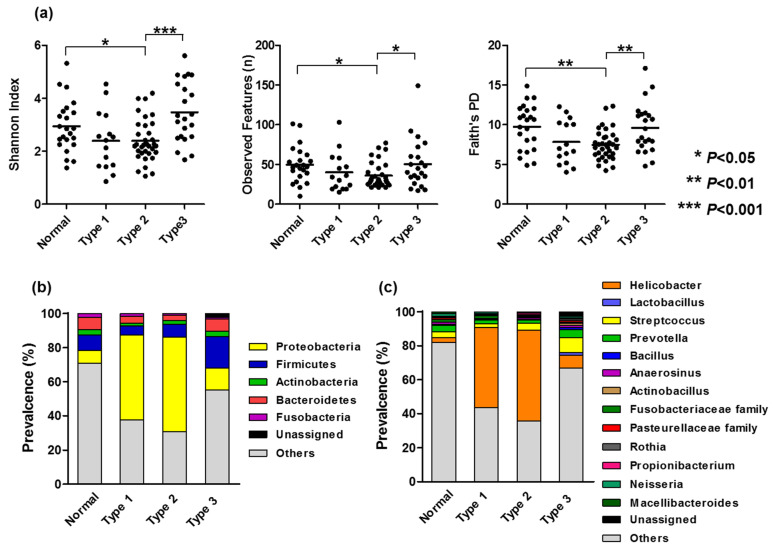
(**a**) Three indicators of alpha diversity measures, Shannon index, observed features and Faith’s phylogenetic diversity (PD) in relation to the gastric mucosal morphologies. Statistical analysis was performed using the Mann–Whitney U test. Overviews of bacterial taxa (>0.5% of relative abundance) at the phylum (**b**) and the family/genus (**c**) levels visualized in relation to the gastric mucosal morphologies.

We first investigated the association between bacterial alpha diversity measures and gastric mucosa morphology using three indicators of alpha diversity measures: the Shannon index, observed features, and Faith’s phylogenetic diversity. This analysis revealed that all three indicators presented a linear decrease in relation to mucosal patterns, from normal to type 1 and 2. However, there was a paradoxical increase between types 2 and 3 (Figure 2a).

Next, we searched for the bacterial taxa associated with gastric mucosal morphology, focusing on phylum and family/genus levels. Bacterial taxa overviews (relative abundance: >0.5%) were visualized in relation to the gastric mucosal morphology (Figure 2b,c), and the list of taxa with significant *p*-values at the family/genus level is listed in Table 1. At the phylum level, the abundance of *Proteobacteria* increased in relation to mucosal patterns, from normal to types 1 and 2, while decreasing considerably in type 3. Conversely, the abundance of *Bacteroides* and *Firmicutes* increased between types 2 and 3. At the family/genus level, *Helicobacter* abundance increased in relation to mucosal patterns, from normal to type 1 and 2, while it decreased markedly in type 3. *Helicobacter* decrease between type 2 and 3 was accompanied by an increase in *Prevotella* and *Streptococcus* (Figure 2b,c). Four specific taxa, whose abundances correlated significantly with mucosal pattern changes from normal to type 1, 2, and 3, were identified at the family/genus level (Table 1). Linear *Streptococcus*, *Actinobacillus* and *Macellibacteroides* increases were associated with mucosal pattern changes, from normal to type 1, 2, and 3 (*Streptococcus*: r = 0.34, *p* = 0.0007; *Actinobacillus*: r = 0.23, *p* = 0.012; *Macellibacteroides*: r = 0.23, *p* = 0.028), while a decrease in the *Fusobacteriaceae family* was associated with mucosal pattern changes from normal to type 1, 2, and 3 (r = −0.24, *p* = 0.019).

**Figure 3:**
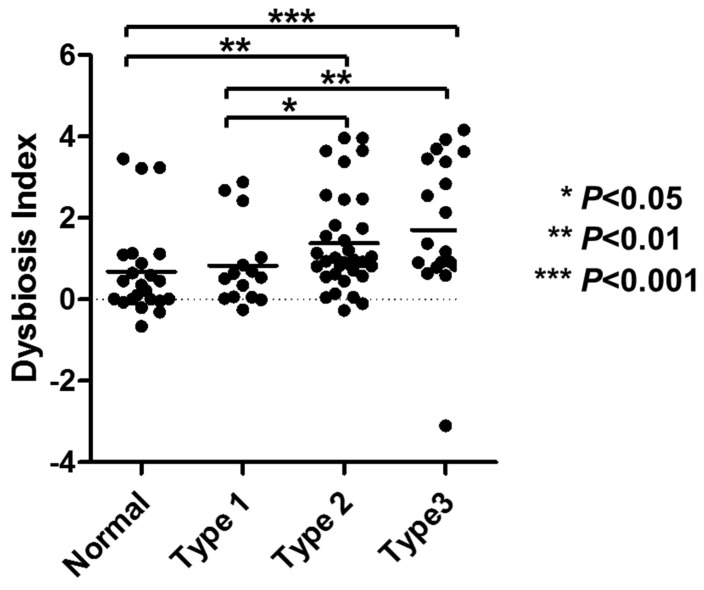
Association between the microbial dysbiosis index and gastric mucosal morphologies. Statistical analysis was performed using the Mann–Whitney U test.

Since synergistic bacterial network contribution to disease has been reported in several diseases [9,21], we combined the relevant taxa to see whether there was a stronger association with gastric mucosa morphologies. To this end, we calculated the microbial dysbiosis index [9,21] by dividing the sum of the abundances of Streptococcus, Actinobacillus, and Macellibacteroides by the abundance of Fusobacteriaceae. This analysis revealed that the linear increase in the dysbiosis index was associated with mucosal pattern changes, from normal to type 1, 2, and 3 (Figure 3).

### 3.3. Association Between Microbiome Dysbiosis and Clinicopathological Characteristics and DNA Methylation

Next, we investigated the association between this dysbiosis index and other clinicopathological characteristics and DNA methylation status (Table 2). This analysis found that a higher dysbiosis index was associated with older age (*p* = 0.02), the severity of inflammation and atrophy based on histology (both *p* = 0.01), gastric cancer occurrence (*p* = 0.0007), and a higher mean DNA methylation Z score (*p* = 0.004). A positive correlation between the higher dysbiosis index and DNA methylation was observed for the nine genes analyzed in this study (Figure S2).

## 4. Discussion

Emerging evidence indicates a substantial involvement of non-*H. pylori* microbiota in gastric carcinogenesis [8,9,22,23]. In insulin–gastrin transgenic mice, a pathogen-free gastric microbiome is associated with more severe gastric premalignant lesions than in those with *H. pylori* infection [22]. In mice, the altered Schaedle’s Flora (a microbiota restricted to the *Clostridium* ASF356, *Lactobacillus murinus* ASF361, and *Bacteroides* ASF519 species) sufficiently promotes gastric atrophy and dysplasia, while coinfection with *H. pylori* led to more severe abnormalities. Indeed, about 70% of the mice with gastric lesions had intraepithelial neoplasia, and the overgrowth of *Lactobacillus murinus* ASF361 correlated with enhanced gastric inflammation and the expression of cancer-related factors, such as TNF-α, Ptger4, and TGF-β [24]. A human case–controlled study comparing patients with gastric cancer with those without cancer identified gastric microbiomes with potential cancer-promoting activities [8,9].

Gastric mucosal morphologies obtained using magnifying NBI can indicate premalignant conditions because of their good correlation with the degree of *H. pylori*-related gastritis [12], gastric cancer occurrence [17], and DNA methylation anomalies [7]. The morphological characterization of gastric mucosae with cancer-promoting microbiomes would be of great interest in view of gastric cancer prevention and high-risk stratification. Therefore, for the first time, we characterized gastric mucosa microbiome communities to determine their association with gastric mucosal morphologies. We also assessed the correlation between the microbiome communities and the DNA methylation anomalies associated with gastric mucosal morphologies.

Our analysis showed decreased microbial alpha-diversity measures in gastric mucosae from the normal to type 1 and 2 groups. However, it paradoxically increased in the type 3 group when compared with the type 2 group. Regarding the association between the gastric morphologies observed using magnifying NBI endoscopy and gastric mucosa histology, NBI mucosal pattern changes from normal to type 1 and 2 correlated with the degree of inflammation, while type 3 was associated with atrophy and metaplasia [12]. We previously reported that the type 3 group is associated with lower ratios of pepsinogen I/II [12], an indicator of the spread of gastric atrophy [24,25]. Since the type 3 group is frequently observed in patients with gastric cancer (Supplementary Table S1 refs. [7,17]), patients with the type 3 pattern can be considered to be at a high risk of developing gastric cancer. A previous study showed that lower bacterial alpha diversity measures in the gastric mucosa were associated with *H. pylori* infection [26,27,28], while studies comparing primary gastric cancer and matched non-cancerous mucosa showed increased alpha diversity measures in primary gastric cancer compared with non-cancerous mucosa [29,30]. These findings suggest that decreased alpha diversity is associated with early microbial change, while an increase in alpha diversity might be relevant for cancer progression. Since decreased alpha diversity measures were paradoxically increased between the type 2 and 3 groups, our findings suggest that microbial changes accompanied by gastric mucosal morphologies represent an initial step towards gastric cancer. The paradoxical change in microbial structures in relation to different gastric morphologies was further characterized by substantial changes in specific bacteria. At the phylum level, the abundance of *Proteobacteria* increased in relation to mucosal patterns, from normal to types 1 and 2, while decreasing considerably in type 3. Conversely, the abundance of *Bacteroides* and *Firmicutes* increased between types 2 and 3. At the family/genus level, the abundance of *Helicobacter* increased in relation to mucosal patterns, from normal to type 1 and 2, while markedly decreasing in type 3. At the family/genus level, there were four specific taxa whose abundance correlated significantly with mucosal pattern changes. Linear increases in *Streptococcus*, *Actinobacillus*, and *Macellibacteroides* abundance and a decrease in the abundance of the *Fusobacteriaceae* family were associated with mucosal pattern changes from normal to type 1, 2, and 3. Importantly, the taxa showing linear increase with mucosal pattern changes included well-known gastric cancer-related bacteria like *Streptococcus*, which was overrepresented in non-neoplastic and neoplastic tissues of patients with gastric cancer [8,9,30]. Coker et al. identified non-*H. pylori* bacteria of oral origin displaying centrality in the GC-related microbial network [8]. Indeed, it is suggested that changes occurring in the stomach following long-term *H. pylori* infection-driven decrease in acid secretion allow the establishment of a new microbiota that contributes to malignant transformation through inflammation maintenance and nitrate conversion into N-nitrosamines [10,11]. Taken together, our results indicate that changes in specific bacteria in relation to gastric morphologies may be an important phenomenon associated with the risk of gastric cancer. We demonstrated that by combining relevant taxa, the microbial dysbiosis index correlated well with mucosal pattern changes from normal to type 1, 2, and 3. We also found that a higher dysbiosis index was associated with older age, inflammation, and atrophy severity based on histology, and the occurrence of gastric cancer. This supports the synergistic contribution of a bacterial network associated with gastric cancer risk, which may also serve as a diagnostic marker, as shown in other studies [9,21]. In this preliminary phase, we used a retrospective design and a small cohort. Whether the dysbiosis index would be useful for gastric cancer risk prediction should be evaluated in various clinical settings. We also demonstrated that the dysbiosis index was associated with molecular anomalies like DNA methylation, suggesting that the non-*H. pylori* bacterial network accelerates carcinogenesis by promoting genotoxic changes. However, our findings indicate correlation without experimental validation of microbial mechanisms. Therefore, well-designed in vivo and ex vivo studies are needed to evaluate how the bacteria identified in our study contribute to gastric tumorigenesis.

## 5. Conclusions

Our results suggest that gastric microbiome communities correlate well with the mucosal morphologies associated with premalignant conditions. Morphological identification of the gastric mucosa associated with the cancer-promoting microbiome would have important clinical implications for gastric cancer prevention. However, since this study was conducted in a single cultural and geographical setting (Japan), the clinical importance of our preliminary findings should be evaluated in other populations with various clinical conditions. *H. pylori* eradication is reported to restore chronic inflammation [31]. The microbial changes accompanied by gastric morphologies after *H. pylori* eradication are also of great interest. On the other hand, these limitations would be of interest for risk stratification and understanding of carcinogenic mechanisms in future studies.

## Figures and Tables

**Table 1:** List of bacteria (genus and family levels), whose abundances were significantly correlated with mucosal pattern changes.

Correlated with the Changes in Mucosal Patterns from Normal to Types 1, 2, and 3.		
Genus and Family	r	*p*-Value
*Streptococcus*	0.34	0.0007
*Actinobacillus*	0.23	0.012
*Fusobacteriaceae family*	−0.24	0.019
*Macellibacteroides*	−0.226	0.028

Statistical analysis was performed using Spearman’s correlation analysis.

**Table 2:** Association between microbial dysbiosis index and clinicopathological factors.

Variables	Dysbiosis Index (+/−SE)	*p*
***Age***		
**<60 y (37)**	**0.98 +/** **− 0.21**	**Reference**
≥**60 y (57)**	**1.34 +/** **− 0.18**	**0.02**
*Gender*		
Male (66)	1.29 +/− 0.17	Reference
Female (28)	0.98 +/− 0.20	0.31
*H. pylori test*		
Negative (41)	1.06 +/− 0.23	Reference
Positive (52)	1.32 +/− 0.17	0.20
***Inflammatory mucosa***		
**Negative (47)**	**1.04 +/** **− 0.19**	**Reference**
**Positive (47)**	**1.36 +/** **− 0.20**	**0.01**
***Atrophic mucosa***		
**Negative (73)**	**1.06 +/** **− 0.14**	**Reference**
**Positive (21)**	**1.68 +/** **− 0.37**	**0.01**
***Cancer occurrence***		
**Cancer-free (71)**	**0.98 +/** **− 0.14**	**Reference**
**Cancer (23)**	**1.86 +/** **− 0.34**	**0.0007**
***Methylation (Z-score)***		
**Low (63)**	**0.99 +/** **− 0.15**	**Reference**
**High (31)**	**1.63 +/** **− 0.28**	**0.004**

Statistical analysis was performed using the Mann–Whitney U test. Significant results are expressed in bold characters. The *H. pylori* test was not performed for 1 patient. Methylation (Z-score) means Z-score of 5 genes (*MYOD1, SLC16A12, GDNF, IGF2, MIR124A1, CDH1, PRDM5, RORA, MLF1*). Methylation-High, mean Z-score methylation ≥−0.3.

## Data Availability

Raw data were generated at Kansai Medical University. The data supporting this study’s findings are available from the corresponding author upon request.

## Supplementary Materials

The following supporting information can be downloaded at: https://www.mdpi.com/article/10.3390/microorganisms13112499/s1, Figure S1: Typical histological findings of gastric mucosa using the HE (hematoxylin-eosin) staining in relation to their NBI gastric mucosal patterns. Normal pattern presents normal mucosa without inflammation. The Type 1 presents mild inflammation. The Type 2 presents severe inflammation with dense mononuclear cell infiltration. The Type 3 presents severe atrophy with intestinal metaplasia; Figure S2: Association between microbial dysbiosis index and DNA methylation status of nine individual genes. Statistical analysis was performed using the Spearman correlation analysis. Table S1: Clinicopathological factors in relation to the normal and types 1 to 3 NBI gastric mucosal patterns.
