# Supplementary material for: The Gastric Microbiome Communities and Endoscopic Mucosal Morphologies Associated with Premalignant Conditions
Source: Microorganisms. 2025 Oct 30;13(11):2499. doi: 10.3390/microorganisms13112499 (PMC12654504; doi:10.3390/microorganisms13112499)
Supplement: Supplementary file 1 [file microorganisms-13-02499-s001.zip › microorganisms-3911767-supplementary.pdf]

Supporting information

Supplementary table S1. Clinicopathological factors in relation to the normal and types 1 to 3 NBI gastric mucosal patterns

Variables	Normal	Type 1	Type 2	Type 3
Total number: n	23	15	34	22
H. pylori positive: n(%)	0 (0%)	13 (86.7%)	34 (100%)	5 (23.8%)
Inflammatory mucosa*: n(%)	0 (0%)	3 (20%)	27 (74.9%)	16 (72.9%)
Atrophic mucosa**: n(%)	0 (0%)	0 (0%)	1 (2.9%)	20 (90.9%)
Gastric cancer: n(%)	0 (0%)	0 (0%)	3 (8.8%)	20 (90.9%)
Z-score of Methylation :mean +/- SD	-1.54 +/- 0.12	-0.60 +/- 0.38	0.11 +/- 0.58	0.26 +/- 0.47

* 2 or higher score of acute or chronic inflammation

** 2 or higher score of atrophy or metaplasia

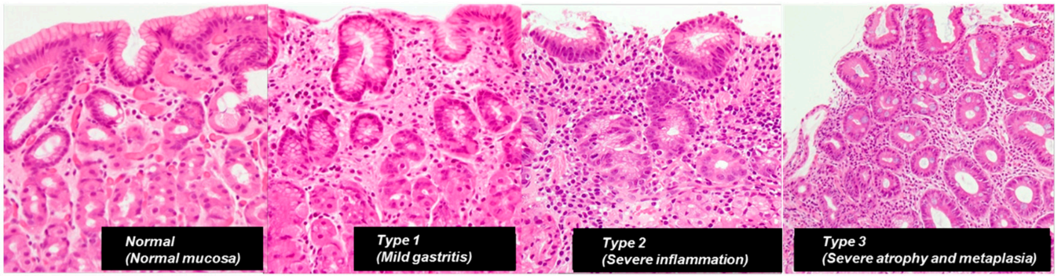

Supplementary Figure S1

Typical histological findings of gastric mucosa using the HE (hematoxylin-eosin) staining in relation to their NBI gastric mucosal patterns. Normal pattern presents normal mucosa without inflammation. The Type 1 presents mild inflammation. The Type 2 presents severe inflammation with dense mononuclear cell infiltration. The Type 3 presents severe atrophy with intestinal metaplasia.

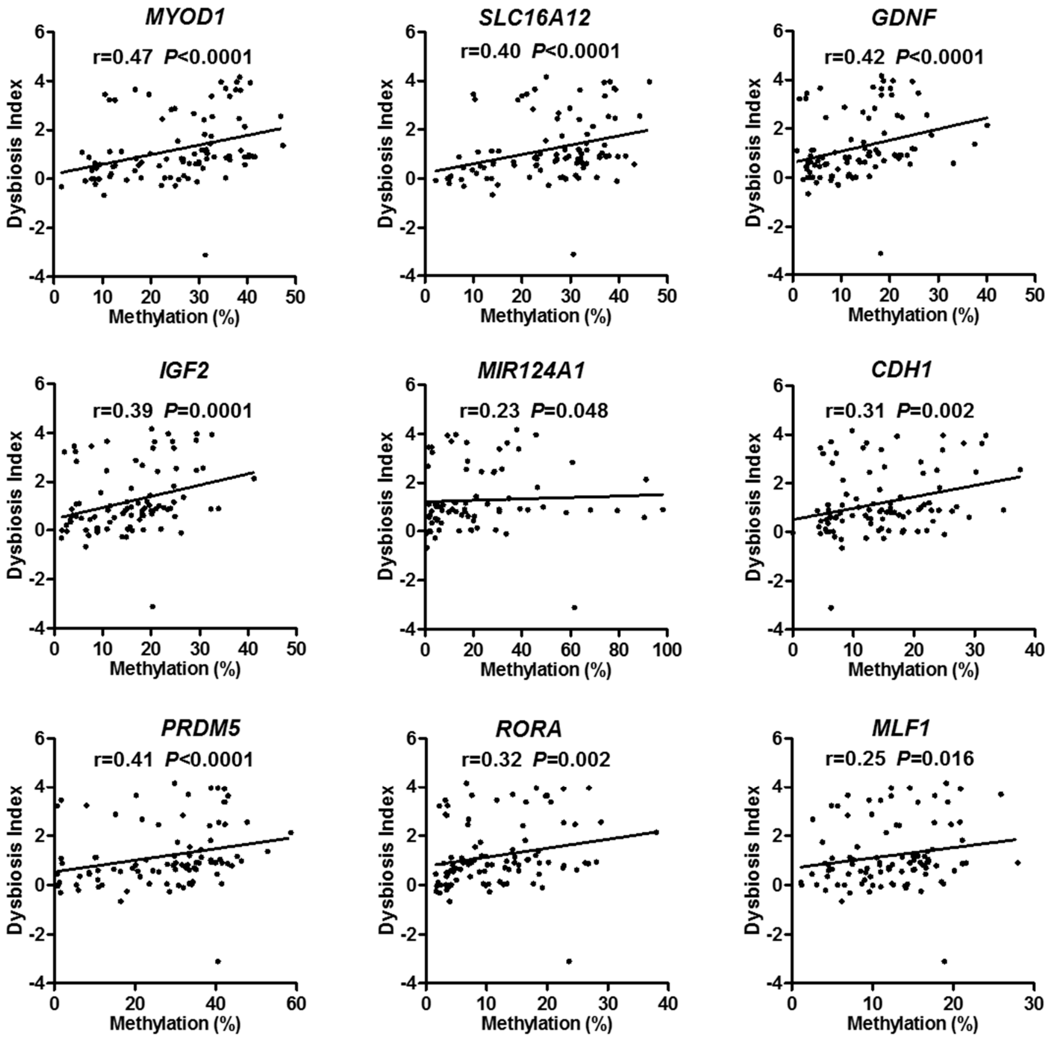

Supplementary Figure S2

Association between microbial dysbiosis index and DNA methylation status of nine individual genes. Statistical analysis was performed using the Spearman correlation analysis.
